# Natural Compounds as Non-Nucleoside Inhibitors of Zika Virus Polymerase through Integration of In Silico and In Vitro Approaches

**DOI:** 10.3390/ph15121493

**Published:** 2022-11-30

**Authors:** Paulo Ricardo Pimenta da Silva Ramos, Melina Mottin, Caroline Sprengel Lima, Letícia R. Assis, Ketllyn Zagato de Oliveira, Nathalya Cristina de Moraes Roso Mesquita, Natasha Marques Cassani, Igor Andrade Santos, Joyce Villa Verde Bastos Borba, Vinícius Alexandre Fiaia Costa, Bruno Junior Neves, Rafael Victorio Carvalho Guido, Glaucius Oliva, Ana Carolina Gomes Jardim, Luis Octávio Regasini, Carolina Horta Andrade

**Affiliations:** 1 LabMol-Laboratory for Molecular Modeling and Drug Design, Faculdade de Farmácia, Universidade Federal de Goiás, Goiania 74605-170, Brazil; 2 Laboratory of Antibiotics and Chemotherapeutics (LAC), Institute of Biosciences, Humanities and Exact Sciences, São Paulo State University (Unesp), Sao José do Rio Preto 15054-000, Brazil; 3 LaBEFar-Laboratory of Structural Biology and Drugs, Institute of Physics of São Carlos, University of São Paulo, Sao Carlos 13563-120, Brazil; 4 Laboratory of Antiviral Research, Institute of Biomedical Science, Federal University of Uberlandia, Uberlandia 38405-302, Brazil

**Keywords:** Zika virus, antiviral, polymerase, docking, drug discovery, NS5 RdRp protein, flavonoid, pedalitin, quercetin, non-nucleoside inhibitor

## Abstract

Although the past epidemic of Zika virus (ZIKV) resulted in severe neurological consequences for infected infants and adults, there are still no approved drugs to treat ZIKV infection. In this study, we applied computational approaches to screen an in-house database of 77 natural and semi-synthetic compounds against ZIKV NS5 RNA-dependent RNA-polymerase (NS5 RdRp), an essential protein for viral RNA elongation during the replication process. For this purpose, we integrated computational approaches such as binding-site conservation, chemical space analysis and molecular docking. As a result, we prioritized nine virtual hits for experimental evaluation. Enzymatic assays confirmed that pedalitin and quercetin inhibited ZIKV NS5 RdRp with IC_50_ values of 4.1 and 0.5 µM, respectively. Moreover, pedalitin also displayed antiviral activity on ZIKV infection with an EC_50_ of 19.28 µM cell-based assays, with low toxicity in Vero cells (CC_50_ = 83.66 µM) and selectivity index of 4.34. These results demonstrate the potential of the natural compounds pedalitin and quercetin as candidates for structural optimization studies towards the discovery of new anti-ZIKV drug candidates.

## 1. Introduction

Zika Virus (ZIKV) is an arthropod-borne flavivirus that circulates globally and caused a worldwide concern due to its exponential spread in the Americas in 2015–2016 [1] and its association with severe congenital effects in pregnant women infected with the virus. The congenital ZIKV syndrome is characterized by neurological and neuropsychomotor complications, ophthalmological and hearing problems, craniofacial disproportion, epilepsy, cerebral palsy and microcephaly [2]. In adults, ZIKV can cause the Guillain-Barre syndrome [3]. Recently, researchers suggested that ZIKV strains with enhanced transmissibility and pathogenicity can reemerge [4]. 

ZIKV is constituted by a single-strand negative RNA which encodes three structural proteins, membrane (M), envelope (E) and capsid protein (C), arranged on a lipidic membrane, and seven non-structural (NS) proteins: NS1, NS2A, NS2B, NS3, NS4A, NS4B and NS5 [5]. Among the NS proteins, the NS5 RNA-dependent RNA-polymerase (RdRp) is an essential protein, catalyzing the replication of viral RNA from the RNA template [6], and has been considered a promising target for ZIKV drug discovery. 

The nucleoside and nucleotide inhibitors (NI) of RdRp bind to the catalytic and RNA binding sites [7], whereas the non-nucleosides inhibitors (NNI) bind to the *N*-pocket (allosteric site) [8]. The NI antiviral drug sofosbuvir has been successfully used against Hepatitis C virus (HCV) and depends on the activation by host kinases [9]. Sofosbuvir was also tested against ZIKV RdRp presenting an IC_50_ value of 0.38 ± 0.03 µM [10]. 

Computer-Assisted Drug Design (CADD) [11] techniques rationally promote the discovery, prioritization and optimization of drug candidates, using computational resources, such as databases, algorithms, programs and web servers. Compared to experimental approaches, such as high-throughput screening (HTS), computational techniques have been shown to be faster and presented higher success rates [12]. 

The present study aimed to discover new potential ZIKV NS5 RdRp inhibitors guided by computational and experimental approaches. DENV and ZIKV NS5 RdRp primary and tertiary sequences share high similarities. Due to this fact, DENV NS5 RdRp known inhibitors were used to search for new potential ZIKV NS5 RdRp hits. Docking calculations were performed to prioritize virtual hits, and enzymatic assays validated these computational predictions, showing that pedalitin and quercetin, two natural compounds, inhibited ZIKV NS5 RdRp. Moreover, both hits presented anti-ZIKV activity in in vitro antiviral assays, with low cytotoxicity. These results demonstrate that integrated in silico and in vitro approaches can be used to accelerate the discovery of new ZIKV antiviral candidates.

## 2. Results and Discussion 

**Figure 1:**
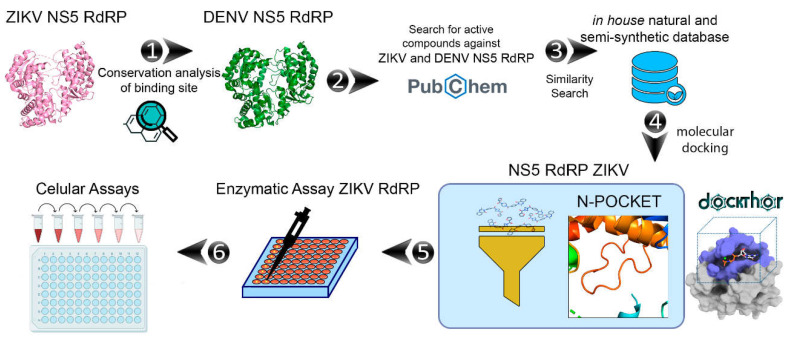
General workflow applied in this work to identify of ZIKV NS5 RdRp inhibitors: (1) conservation analysis of ZIKV NS5 RdRp; (2) collection of compounds with experimental data against DENV and ZIKV RdRp in the PubChem database; (3) similarity analysis between known RdRp inhibitors and in-house collection of untested compounds available on the Laboratory of Antibiotics and Chemotherapeutics (LAC); (4) molecular docking of prioritized compounds at the ZIKV NS5 RdRp *N*-pocket binding site; (5) enzymatic and (6) cellular assays.

A general workflow of the computational and experimental steps applied in this study is presented in Figure 1. 

### 2.1. Binding Site Conservation Analysis

**Figure 2:**
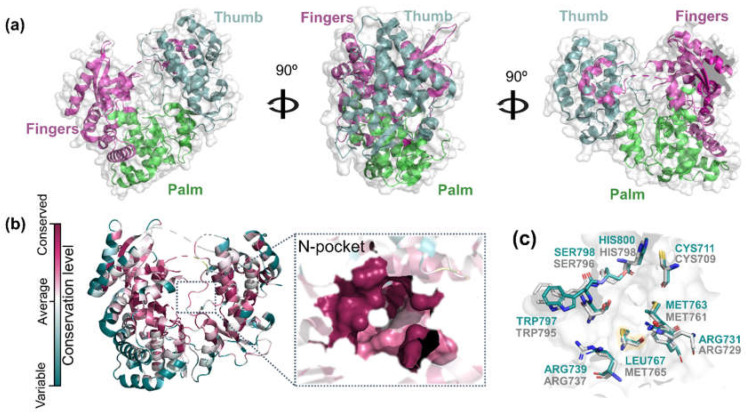
Structural analysis of RdRp proteins from ZIKV (PDB ID: 6LD4) and DENV (PDB ID: 5I3Q). (**a**) Cartoon backbone diagram showing the front, side and back views of ZIKV RdRp. The fingers, thumb and palm domains are colored in magenta, cyan and green, respectively. (**b**) ConSurf analysis of ZIKV RdRp and corresponding *N*-pocket site. The magenta color indicates high conservation while white and turquoise colors indicate average and very low conservation, respectively. (**c**) Structural overlap of ZIKV and DENV *N*-pocket sites. ZIKV and DENV residues are colored in cyan and gray, respectively.

The binding-site conservation can provide an invaluable resource to understanding the affinity and binding mode of small molecules between homologs. In theory, proteins sharing a high similarity have the probability of sharing the same ligands [13]. Here, we employed the ConSurf analysis [14,15,16] to predict the evolutionary conservation profile of ZIKV RdRp amino acids based on the phylogenetic relations between homologous sequences such as DENV RdRp. All the polymerases resemble a right hand, with the three main regions (Figure 2a): fingers (residues 321–488 and 542–608), palm (residues 489–541 and 609–714), and thumb (residues 715–903). The RdRp domain is composed of three binding sites: the RNA site, the *N*-pocket (allosteric site) and the catalytic binding site [7,8,17]. The RNA site is a tunnel that single-stranded RNA enters and serves as a template for the formation of double-stranded RNA. The *N*-pocket is a tunnel through which the nucleotides enter. At this site, the initiation loop regulates template RNA binding and nucleotide entry. Finally, the catalytic site performs double-stranded RNA catalysis [18]. 

Although the DENV and ZIKV RdRp proteins show 64.59% of sequential identity, the evolutionary analysis of viral RdRps shows that ZIKV *N*-pocket is highly conserved (Figure 2b). These results suggest that the amino acid composition of the *N*-pocket is strongly associated with its structural and functional importance. As we can see in Figure 2c, all DENV (highlighted in gray, PDB ID: 5I3Q [18]) and ZIKV (highlighted in cyan, PDB ID: 6LD4 [19]) *N*-pocket residues are conserved, except for Leu767 in ZIKV, replaced by Met765 in DENV RdRp. It is important to point out that these two amino acids share similar volumes and electronic properties, and thus should not promote significative changes in the binding of small molecules to the *N*-pocket. The high conservation state of *N*-pockets corroborates with a high probability of ZIKV and DENV RdRps to share the same ligands. Based on these findings, an unsupervised cheminformatics approach using known DEV RdRp inhibitors was performed to find ZIKV RdRp hits from an in-house collection of natural and semi-synthetic compounds.

### 2.2. Chemical Space Analysis of RdRp Inhibitors

A chemical space analysis was conducted in order to select compounds from our in-house library that are similar to known RdRp inhibitors. Therefore, we compiled a dataset of known DENV RdRp inhibitors from PubChem and the literature. In total, 94 compounds were obtained from several bioassays on PubChem AID: 441537 [20], 642356 [21], 663478 [22], 1277364 [23], 1301573 [24], 1401288 [25], 1401306 [25], 1497239 [26], 1655471 [27], 1674514 [28], 1728708 [29] and 30 compounds were manually collected from published studies [17,20,21,23,25,27,28,30,31,32,33,34,35,36,37,38,39,40,41], providing a dataset of 124 DENV RdRp inhibitors. The in-house dataset from the Laboratory of Antibiotics and Chemotherapeutics (LAC), at São Paulo State University (UNESP), presents 77 natural and semi-synthetic compounds was merged to the publicly available dataset and a chemical space analysis was conducted using the dimensionality reduction method t-Distributed Stochastic Neighbor Embedding (t-SNE) [42].

**Figure 3:**
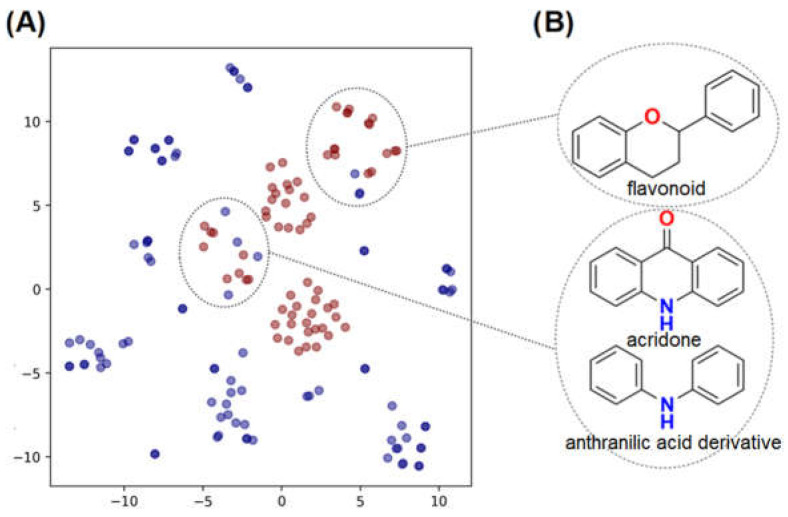
t-SNE plot showing the chemical space of known DENV RdRp inhibitors and our in-house database of natural and semi-synthetic compounds. (**A**) DENV RdRp inhibitors collected from the literature and PubChem are shown in blue circles. The *in house* natural and semi-synthetic database compounds are represented in red. (**B**) 2D structures of the main scaffolds found in each cluster.

As shown in the t-SNE plot (Figure 3A), 24 compounds from the in-house collection share the same chemical space of the known NS5 RdRp inhibitors. Most of them belong to the classes of acridones, diphenylamines, and flavonoids (Figure 3B). In view of this, these compounds were prioritized for molecular docking to assess their binding modes in ZIKV RdRp protein [19]. Since the scaffolds of the compounds are different from the nucleoside-like structure, the analysis was focused on the *N*-pocket site. 

### 2.3. Docking Calculations at the ZIKV NS5 RdRp (N-Pocket) 

The prioritized compounds from chemical space analysis were submitted to docking calculations to rank the most promising hits as well as to predict the binding affinities. All docking poses were analyzed according to the following parameters: (*i*) docking score and intermolecular interactions at the *N*-pocket binding site; (*ii*) overlap and binding mode similarity with the ZIKV RdRp NNI co-crystallized ligand 5-(3-fluorothiophen-2-yl)-2-hydroxy-4-methoxy-N-[4-(trifluoromethyl)benzenesulfonyl]benzamide and (*iii*) ligand efficiency. The redocking of the co-crystallized ligand was performed to verify the accuracy of the docking protocol in predicting the position of the ligands within the binding site (Supplementary Figure S2). Redocking also gives us a reference value of the docking score to consider in the compounds’ priorization. The redocked pose showed an RMSD value of 0.84 Å and a docking score value of −8.73 Kcal·mol^−1^.

Almost all ligands presented acceptable docking scores, close to the redocking score of the co-crystalized compound (docking score −8.73 Kcal·mol^−1^). Moreover, analyzing the binding modes and interactions, 16 ligands were prioritized for in vitro experimental validation. Thirteen of them had similar binding modes with the known ZIKV RdRp NNI. From them, 12 compounds presented ligand efficiency (LE) greater than 0.3 Kcal·mol^−1^·non-hydrogen atom^−1^. LE is value that normalizes the binding affinity (ΔG) or docking score with respect to the number of non-hydrogen atoms (*n*) [43,44,45]. The normalization of molecular weights influences the likelihood that a hit compound can be further optimized into prospective hit-to-lead investigations, as larger compounds tend to show greater docking scores due to the larger number of interactions [46,47].

A medicinal chemistry-based inspection was conducted [47,48], considering favorable scores for a higher number of hydrogen bonds between ligand and protein residues; salt bridges; π-cation and π-stacking interactions; and unfavorable scores for nonpolar regions of the ligand exposed to solvent. After this inspection, nine compounds were prioritized for the experimental evaluation (Table 1).

**Figure 4:**
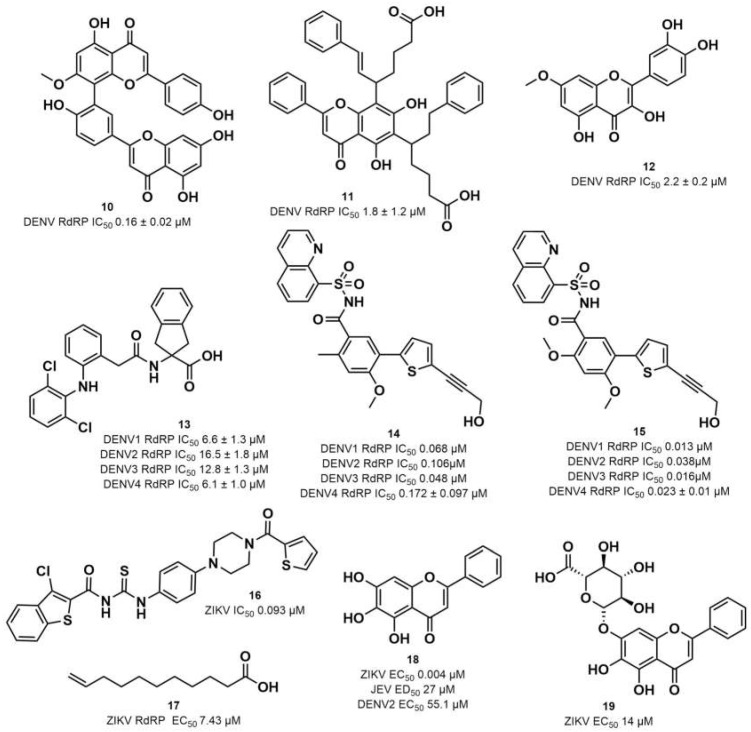
DENV and ZIKV RdRp known inhibitors and their respective IC_50_ values at RdRp: (**10**) podocarpusflavone A [31]; (**11**) chartaceone D [21]; (**12**) rhamnetin [30]; (**13**) bis-chloro-diphenylamine, 2-aminoindan-2-carboxyl derivative [49]; (**14**) 5-(5-(3-Hydroxyprop-1-yn-1-yl)thiophen-2-yl)-2,4-dimethoxy-*N*-((3-methoxyphenyl)sulfonyl)benzamide [8]; (**15**) 5-(5-(3-Hydroxyprop-1-yn-1-yl)thiophen-2-yl)-4-methoxy-2-methyl-*N*-(quinolin-8-ylsulfonyl)benzamide [24]; (**16**) TPB [57]; (**17**) undecylenic acid [38]. Flavones (**18**) baicalein [59,60,61] and (**19**) baicalin [59] with antiviral activity (EC_50_) against several flaviviruses.

Four out of the nine virtual hits are naturally-occurring flavonoids (chrysin (**6**), sorbifolin (**7**), pedalitin (**8**) and quercetin (**9**)). Flavonoids have already been described in the literature as inhibitors of the RdRp domains of DENV and ZIKV [49]. Three compounds belong to the class of acridones, a class already described by some authors due to their antiviral activity and capability of inhibition of DNA and RNA viruses [50,51]. A potent activity of *N*-substituted acridones has already been demonstrated against DENV-2, blocking its multiplication in vitro [52]. ARORA and coworkers [53] demonstrated that compounds containing the diphenylamine subunit were able to inhibit the RdRp domain of DENV including the compound bis-chloro-diphenylamine, 2-aminoindan-2-carboxyl derivative NITD-434 (**13**) (Figure 4) that interacts with residues Thr795 and Thr796 of the *N*-pocket site. Three of the nine hits are diphenylamines. 

RdRp inhibitors have been classified as NI and NNI. The NIs present a structural similarity to nucleosides and have to be converted into triphosphate forms by host kinases to be incorporated into viral DNA or RNA, acting as chain terminators [54]. On the other hand, the NNIs interact directly with viral polymerase and present different scaffolds, such as flavonoids, alkaloids, acetylenic acids, terpenes, steroids, benzothiazine 2,2-dioxide analogs, pyrazole-5-phenylamine analogs, thiophene-based analogs, *N*-sulfonylpyrazoles and *N*-sulfonylanthranilic acids, thiazolidinone-thiadiazole and pyridobenzothiazole analogs [49]. NNIs act into the RdRp allosteric site and, in general, display fewer side effects since they are more selective for viral than host polymerase targets [55]. Among the DENV NNIs, there are natural products including flavonoids **10, 11** and **12** (Figure 4). Furthermore, another DENV RdRp NNI, the bis-chloro-diphenylamine, 2-aminoindan-2-carboxyl derivative compound (**13**) or NITD-434 (Figure 4), occupies the template RNA site and performs interactions with conserved residues between the four serotypes of DENV and ZIKV [53]. The synthetic co-crystallized DENV RdRp NNIs acylsulfonamide derivatives compounds (**14**) and (**15**) [8] (Figure 4), occupy the *N*-pocket site and had IC_50_ values ranging from 0.172 to 5.46 μM for compound **14** and 0.023 to 0.427 μM for compound **15** [10,56]. Among the ZIKV NNIs, there are few natural compounds such as chalcones and alkaloids, as well as synthetic compounds undecylenic acid compound **17** (Figure 4) and thienylcarbonyl-piperazinyl-benzothiophene (TBP), compound **16** (Figure 4) that act to inhibit ZIKV NS5 RdRp [57].

Recently, the flavonoids luteolin and quercetin were tested against severe acute respiratory syndrome coronavirus 2 (SARS-CoV-2) RdRp and presented IC_50_s values of 4.6 µM and 6.9 µM, respectively [58]. The authors also performed docking and molecular dynamic simulations of both ligands at the *N*-pocket and RNA binding sites, suggesting that they may properly bind to both sites.

### 2.4. Pedalitin and Quercetin Inhibits ZIKV RdRp Activity 

Nine prioritized hits were submitted to endpoint assay at 20 µM to verify their inhibitory activity against ZIKV RdRp. Most of the compounds evaluated did not obtain significant inhibition results. Pedalitin and quercetin were the only ones with activity greater than 80%, with inhibitory activity of 97% and 99%, respectively, and consequently were selected for the concentration-response assays.

We then investigated ZIKV RdRp activity in the presence of pedalitin and quercetin. A concentration-response assay was performed at concentrations ranging from 80 µM to 0.156 µM to determine the inhibitory concentration of 50% (IC_50_). From this range of concentrations, it was determined that pedalitin and quercetin had IC_50_ values of 4.1 ± 0.3 µM and 0.5 ± 0.1 µM, respectively (Supplementary Figure S1). The enzymatic activities obtained are in agreement with those described for other flavonoids, as shown in Figure 4. 

### 2.5. Pedalitin and Quercetin Binding Modes Predicted by Docking

**Figure 5:**
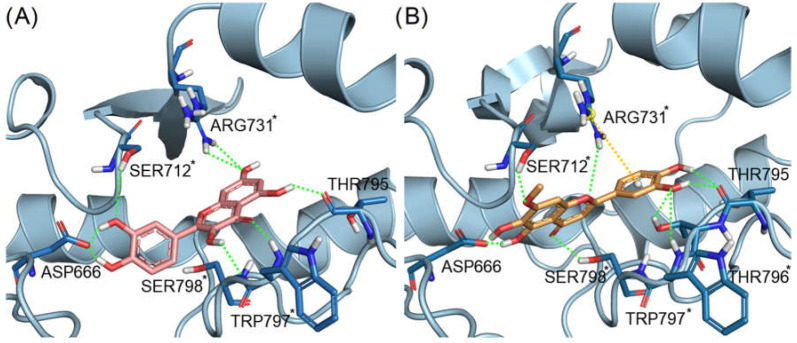
Docking poses of (**A**) quercetin (carbon atoms in pink sticks representation) and (**B**) pedalitin (carbon atoms in orange sticks) at the *N*-pocket of ZIKV RdRp. Hydrogen bonds are represented as green dotted lines and cation–π interactions in yellow dotted lines. The interactions of residues highlighted with an asterisk (*) are the same observed with the co-crystallized ligand.

From the enzymatic data, the two flavonoids pedalitin and quercetin were highlighted as promising ZIKV RdRp inhibitors. In Figure 5A,B we show the binding mode of quercetin and pedalitin, predicted by our docking calculations. Quercetin presented four interactions highlighted with an asterisk (*) (Figure 5A), that are the same interactions performed by the co-crystallized 5-(3-fluorothiophen-2-yl)-2-hydroxy-4-methoxy-N-[4-(trifluoromethyl)benzenesulfonyl]benzamide compound. These interactions are hydrogen bonds with residues Ser712, Arg731, Trp797, Ser798 and Asp666 (catalytic triad residue). In the same way, pedalitin presented four hydrogen bonds, with Ser712, Ser798, Trp797 and Thr796 and a cation–π interaction with the residue Arg731. Moreover, it also interacts with Asp666 via a hydrogen bond. 

The binding modes of the flavonoids quercetin and pedalitin predicted by docking with ZIKV RdRp suggested a promising binding affinity with the allosteric binding site of the protein, corroborating the enzymatic assays results.

### 2.6. Pedalitin and Quercetin Inhibits ZIKV Replication In Vitro 

**Figure 6:**
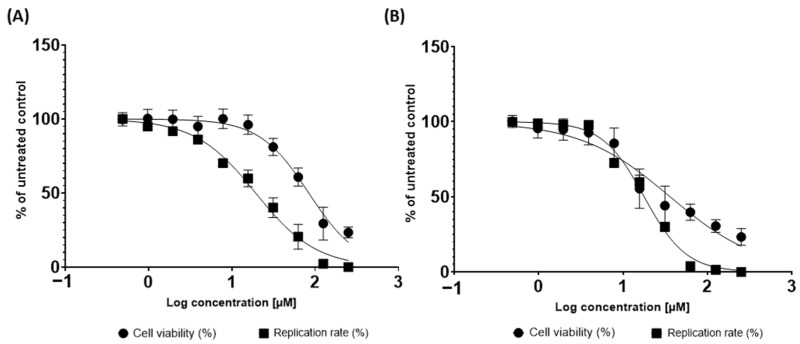
Inhibition of replicative viral cycle by activity of pedalitin (**A**) and quercetin (**B**) against ZIKV^BR^ infection. ZIKV^BR^ replication was evaluated by measuring focus formation units using an immunofluorescence assay (indicated by ▄) after 72 h.p.i.. Cellular viability was measured in parallel using an MTT assay (indicated by ●). Mean values of three independent experiments each measured in quadruplicate including the standard deviation are shown.

The anti-ZIKV activities of the pedalitin and quercetin were further investigated through the employment of Vero cells infected with ZIKV wild type (ZIKV^BR^) (Figure 6). For this, a concentration-response assay was performed to determine the effective concentration of 50% (EC_50_) and cytotoxicity of 50% (CC_50_), and to calculate the Selective Index (SI = CC_50_/EC_50_). Vero cells were infected with ZIKV^BR^ and simultaneously treated with pedalitin or quercetin at concentrations ranging from 200 µM to 0.005 µM for 72 h when viral replication rates were assessed (Figure 6). Cell viability analysis was performed in parallel (Figure 6).

From this range of concentrations, the treatment of ZIKV-infected cells with pedalitin demonstrated an EC_50_ value of 19.28_,_ CC_50_ value of 83.66, and SI value of 4.34, and quercetin demonstrated an EC_50_ value of 17.74_,_ CC_50_ value of 35.99, and SI value of 2.03 (Table 2). 

Summarizing computational and experimental data (Table 2), both pedalitin and quercetin bound to the *N*-pocket site of ZIKV RdRp, presenting good docking scores, compared to the redocking calculations, and binding site interactions similar with the co-crystalized ligand. Agreeing with docking calculations, enzymatic assays showed that both flavonoids inhibited ZIKV RdRp activity. Moreover, infection assays demonstrated that both compounds presented in vitro antiviral activity, and pedalitin presented a higher selectivity index (SI), representing a more promising hit.

Other flavonoid compounds have already demonstrated anti-ZIKV effects on Vero cells [62], such as the flavones baicalein (**18**) and baicalin (**19**) (Figure 4), which showed an EC_50_ of 0.004 µM and 14 µM, respectively [59]. Baicalein was also tested against other flaviviruses, such as Japanese encephalitis virus (JEV) and DENV-2, displaying an EC_50_ values of 27 ± 4 µM [52] and 55.1 µM, respectively [60,61]. Quercetin, identified in our study, also demonstrated anti-viral activity against DENV-2 virus in the study of Zandi and coworkers [63]. They used DENV-2 infected Vero cells and tested different concentrations of quercetin. At a concentration of 165.4 µM, the replication was reduced by 67%. Concentration-response curves were performed with administration after viral adsorption to the cells, obtaining an EC_50_ value of 95.6 µM [63]. 

Alternatively, there is a crucial role in virus-host cell interactions that provide important targets for the development of non-specific acting antivirals [64]. Non-specific antivirals can interfere with viral infection by acting on cellular signaling pathways or by modulating the differentiation and function of several immune cells [65]. This antiviral effect might contribute to a lower probability to develop viral resistance due to their reliance on host cell components [64]. Additionally, the intervention of virus-host interactions can include a broader range of activity with the immune system, especially for unknown emergent viral infections, where replication mechanisms are not elucidated [66]. 

Flavivirus polymerases have been reported to antagonize the interferon (IFN) signaling pathway via numerous mechanisms, including STAT2 degradation, inhibition of RIG-I, and suppression of IFNAR1 maturation [67,68,69]. Combating flavivirus infections by modulating the signaling pathway could be a factor to improve the infection outcome. In this case, non-specific antivirals are particularly desirable to be used combined with direct-acting antivirals and prepare the scientific community for future epidemics [70]. 

## 3. Materials and Methods 

### 3.1. Computational

#### 3.1.1. DENV and ZIKV NS5 RdRp Similarity Analysis

The 3D structure of ZIKV NS5 RdRp (PDB ID: 6LD4 [19]) was submitted to the ConSurf server [14,15,16] for estimating the evolutionary conservation of amino acids, based on their phylogenetic relations with homologues. Initially, 150 homologue sequences were imported from UNIREF-90 database [71]. The sequences with sequential identity <35% or >95% were ignored. A multiple sequence alignment (MSA) of the homologous sequences was built using the MAFFT-L-INS-I method [72] and the phylogenetic tree was built using the neighbor-joining algorithm [73]. Position-specific conservation scores were then computed using the empirical Bayesian method [74]. At the end of this analysis, the 3D structures and FASTA sequences of ZIKV (PDB ID: 5I3Q [8]) and DENV (PDB ID: 6LD4 [19]) were aligned using the PyMol v. 2.4 [75] and UniProt [76], respectively. The root-mean-square deviation (RMSD) was calculated for the distances of the conserved residues.

#### 3.1.2. Collection of DENV RdRp Inhibitors

Initially, a search was performed in PubChem [77,78,79,80] databases for inhibitors of the NS5 RdRp of DENV. The activity IC_50_ threshold for component selection was 50 µM defined by PubChem. The bioassays selected for RdRp data collection were: PubChem AID: 441537 [20], 642356 [21], 663478 [22], 1277364 [23], 1301573 [24], 1401288 [25], 1401306 [25], 1497239 [26], 1655471 [27], 1674514 [28] and 1728708 [29]. Moreover, RdRp inhibitors from articles were manually collected from the literature [17,20,21,23,25,27,28,30,31,32,33,34,35,36,37,38,39,40,41] and added to our database.

#### 3.1.3. Chemical Space Analysis of RdRp Inhibitors

The chemical space of known DENV RdRp inhibitors and an in-house collection of natural and semi-synthetic compounds was performed using t-Distributed Stochastic Neighbor Embedding (t-SNE) [42]. The t-SNE dimensionality reduction was performed using scikit-learn v. 1.0.2 [81] and extended connectivity fingerprints (ECFP6) with 2048 bits available on RDkit package v. 2022.03.2 [82].

#### 3.1.4. Protein and Ligand Preparation 

The 3D structure of ZIKV NS5 RdRp (PDB ID: 6LD4; resolution: 1.5 Å [19]) complexed with the compound 5-(3-fluorothiophen-2-yl)-2-hydroxy-4-methoxy-N-[4-(trifluoromethyl)benzenesulfonyl]benzamide was imported into Maestro workspace v.9.3 (Schrödinger, LCC, New York, NY, USA, 2012) and processed using the Protein Preparation Wizard [83,84]. In this step, bond orders and formal charges were adjusted, while hydrogen atoms were added to the protein. The protonation states (pKa) of polar amino acid residues were predicted by the Epik program [85,86] at pH = 7.4 ± 0.5, whereas the OPLS-2005 force field was used to minimize the energy of the 3D structure. In parallel, protonation states and 3D geometric optimization of prioritized compounds were predicted using LigPrep software [84,87] at pH = 7.4 ± 0.5.

#### 3.1.5. Molecular Docking 

Molecular docking studies were performed using the DockThor VS webserver [88,89]. The grid box was centered at the *x*, *y*, and *z* coordinates of the co-crystallized ligand bound to the *N*-pocket site. The search algorithm precision mode was set up as the standard configuration of genetic algorithm parameters, and the soft docking mode was activated. At the end of the docking procedure, we used the PLIP server [90] to analyze the intermolecular interaction patterns of the docking poses (hydrogen bonds, hydrophobic interaction, cation-π, π-stacking, water and salt bridge interactions). Finally, the binding mode of the ligands obtained was compared to that of co-crystallized ligand (PDB ID: 6LD4). Then, the Pymol software v. 2.4 [75] was used for visual inspection and to render the pose images.

### 3.2. Experimental

#### 3.2.1. Quercetin and Pedalitin

Quercetin and pedalitin were obtained from *Pterogyne nitens*, a medicinal Brazilian tree, according to our previous phytochemical procedures [91].

#### 3.2.2. Protein Cloning, Expression and Purification 

ZIKV NS5 RdRp polymerase was cloned at pETTRX by the LIC method and expressed and purified according to the protocol described in [92]. Briefly, NS5 RdRp polymerase was expressed in ZYM 5052 auto-induction medium and purified in four steps: (*i*) a HisTrap HP 5.0 mL with a Ni Sepharose resin (GE Healthcare, Sao Carlos, Brazil); (*ii*) a buffer exchanged by dialysis and a concomitant TEV protease cleavage from 6His-TRX-tag; (*iii*) an inverse HisTrap HP 5.0 mL to separate protein from 6His-TRX-tag and (*iv*) a size-exclusion chromatography at a XK 16/60 Superdex 75 column (GE Healthcare, Sao Carlos, Brazil). 

#### 3.2.3. NS5 RdRp Activity Assays

ZIKV NS5-RdRp activity assays were performed as described by Fernandes and coworkers [93]. The endpoint assays were performed at 20 μM, and the compounds that inhibited more than 80% of activity in this assay were submitted to a concentration-response test. The concentration-responses assays were performed as described in [94]. In all cases, the percentage inhibition values were calculated based on a control reaction, containing only DMSO in the same concentrations used for the tested compounds. The results were analyzed and plotted using the GraphPad Prism v. 8.0 program [95].

### 3.3. Cell Culture 

Vero cells were cultured in Dulbecco’s modified Eagle’s medium (DMEM; Sigma–Aldrich, MO, USA) supplemented with 100 U/mL penicillin (Gibco Life Technologies, Paisley, UK), 100 mg/mL streptomycin (Gibco Life Technologies, Paisley, UK), 1% (*v*/*v*) non-essential amino acids (Gibco Life Technologies, Paisley, UK) and 10% (*v*/*v*) fetal bovine serum (FBS; Hyclone, UT, USA) at 37 °C in a humidified 5% CO_2_ incubator.

### 3.4. Virus Rescue and Titration

A wild-type ZIKV isolate from a clinical patient in Brazil (ZIKV^BR^, PA, Brazil) was provided by the Evandro Chagas Institute in Belém, Pará [96]. The virus was amplified employing Vero cells in a 175 cm^2^ flask. To determine viral titers, 1 × 10^4^ Vero cells were seeded in each of 24 wells plate 24 h prior to the infection. Cells were infected with 10-fold serially dilutions of ZIKV^BR^ for 1 h at 37 °C and then supplemented with medium containing 1% penicillin, 1% streptomycin, 2% FBS and 1% carboxymethyl cellulose (CMC). Infected cells were incubated for seven days in a humidified 5% CO_2_ incubator at 37 °C, followed by fixation with 4% formaldehyde and staining with 0.5% violet crystal. The viral foci were counted to determine viral titers which were expressed in plaque formation unit per milliliters (PFU/mL).

### 3.5. Cell Viabillity

Cell viability was measured by the MTT [3-(4,5-dimethylthiazol-2-yl)-2,5-diphenyl tetrazolium bromide] (Sigma–Aldrich) method. Vero cells were seeded in a 96-well plate at a density of 5 × 10^3^ cells per well and incubated overnight at 37 °C in a humidified 5% CO_2_ incubator. A drug-containing medium at concentrations ranging from 200 to 0.005 µM was added to the cell culture. After 72 h at 37 °C, the media was removed and a solution containing MTT at the final concentration of 1 mg/mL was added to each well and incubated for 30 min at 37 °C in a humidified 5% CO_2_ incubator, after which media was replaced with 100 μL of DMSO to solubilize the formazan crystals. Absorbance was measured by the optical density (OD) of each well at 490 nm, using a spectrophotometer. Cell viability was calculated according to the equation (T/C) × 100%, where T and C represent the mean optical density of the treated group and vehicle control group, respectively. The cytotoxic concentration of 50% (CC_50_) was calculated using Graph Pad Prism v. 8 [95].

### 3.6. Antiviral Assays

Vero cells were seeded at density of 5 × 10^3^ cells per well into 96-well plates 24 h prior to the infection. ZIKV-WT^BR^ at a multiplicity of infection (MOI) of 0.1 and compound at concentrations ranging from 200 to 0.005 µM were simultaneously added to cells. 72 h post-infection (h.p.i.), cells were fixed with paraformaldehyde 4%, washed with PBS and blocking buffer (BB) containing: 0.1% Triton X-100 (Vetec Labs, PR, BR), 0.2% bovine albumin (BSA) and PBS for 30 min. Then, cells were incubated with primary rabbit polyclonal anti-NS3 antibody diluted in BB for 1 h. Alexa Fluor 488 conjugated anti-rabbit IgG was used as secondary antibody (Abcam, Cambridge, UK). Images were analyzed by EVO cell imaging systems fluorescence microscopy (Thermo Fisher Scientific, OH, USA) and foci of infection were counted. The antiviral activity was calculated according to the equation (T/C) × 100%, where T and C represent the mean of the treated group and mean of the last concentration, respectively. The effective concentration of 50% inhibition (EC_50_) was calculated using Graph Pad Prism v. 8. The values of CC_50_ and EC_50_ were used to calculate the selectivity index (SI = CC_50_/EC_50_).

## 4. Conclusions

Despite the severe neurological consequences caused by ZIKV infection, there is still no antiviral for the treatment of ZIKV, and only a few ZIKV NS5 RdRp inhibitors have been described in the literature. In our study, guided by known DENV NS5 RdRp inhibitors, through binding site conservation and chemical space analysis as well as docking calculations we prioritized and identified the flavonoids pedalitin and quercetin as new inhibitors of ZIKV NS5 RdRp. Enzymatic assays reinforced the computational results, and both compounds presented antiviral activity against ZIKV in infected cell cultures. Therefore, quercetin and pedalitin may be promising candidates for hit-to-lead optimization, boosting the discovery of new anti-ZIKV drug candidates.

## Figures and Tables

**Table 1:** Virtual hits prioritized based on the computational approaches.

Compound	Structure	Chemical Class	Docking Score (Kcal·mol^−1^)	LE * (Kcal·mol^−1^·Non-Hydrogen Atom^−1^)
**1**	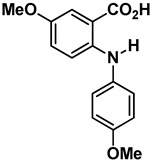	Anthranilic acid derivative	−8.12	0.43
**2**	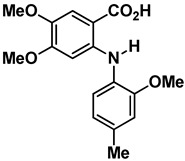	Anthranilic acid derivative	−8.43	0.38
**3**	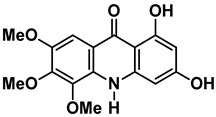	Acridone	−8.68	0.38
**4**	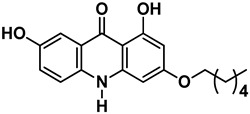	Acridone	−8.30	0.35
**5**	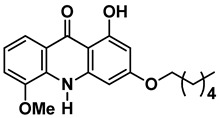	Acridone	−8.72	0.35
**6** **Chrysin**	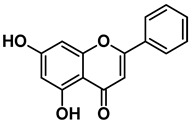	Flavonoid	−8.14	0.43
**7** **Sorbifolin**	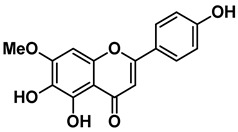	Flavonoid	−8.36	0.38
**8** **Pedalitin**	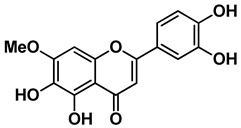	Flavonoid	−7.94	0.35
**9** **Quercetin**	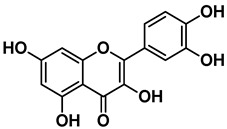	Flavonoid	−7.74	0.35

* LE = docking score*(# of heavy atoms)^−1^.

**Table 2:** Summary of the computational and experimental results for the best two compounds found in this study.

Compound	Docking Score (Kcal·mol^−1^)	IC_50_ ZIKV RdRp (µM)	EC_50_ ZIKV (µM)	CC_50_ (µM)	SI *
pedalitin	−7.93	4.1 ± 0.3	19.28	83.66	4.34
quercetin	−7.74	0.5 ± 0.1	17.74	35.99	2.03

* Selectivity index, SI = CC_50_/EC_50._

## Data Availability

Data is contained within the article and Supplementary Material.

## Supplementary Materials

The following supporting information can be downloaded at: https://www.mdpi.com/article/10.3390/ph15121493/s1. Figure S1: ZIKV NS5 RdRp enzymatic assays. Concentration-response curves adjusted with Hill to determine IC50 ± Δ IC50 values for (A) pedalitin and (B) quercetin. Figure S2: Superposition of the co-crystallized 5-(3-fluorothiophen-2-yl)-2-hydroxy-4-methoxy-N-[4-(trifluoromethyl)benzenesulfonyl]benzamide compound in crystal (C atoms are represented in blue) and the redocking pose (C atoms are represented in green) at the ZIKV NS5 RdRP structure (PDB ID 6LD4 [21]). Table S1: Docking results for all compounds selected by chemical space analysis.

## Abbreviations

DENV: Dengue virus; HCV, Hepatitis C virus; NI, nucleoside and nucleotide inhibitor; NNI, non-nucleoside inhibitor; capsid; M, membrane; E, envelope; NS, nonstructural proteins; RdRp, RNA-dependent RNA-polymerase; ZIKV, Zika virus.
