# Supplementary material for: Natural Compounds as Non-Nucleoside Inhibitors of Zika Virus Polymerase through Integration of In Silico and In Vitro Approaches
Source: Pharmaceuticals (Basel). 2022 Nov 30;15(12):1493. doi: 10.3390/ph15121493 (PMC9788182; doi:10.3390/ph15121493)
Supplement: Supplementary file 1 [file pharmaceuticals-15-01493-s001.zip › pharmaceuticals-2025085-supplementary.pdf]

Supplementary Material for

Natural compounds as non-nucleoside inhibitors of Zika virus polymerase through integration of *in silico* and *in vitro* approaches

Paulo Ricardo Pimenta da Silva Ramos[1‡]; Melina Mottin[1‡]; Caroline Sprengel Lima[2]; Letícia R. Assis[2]; Ketllyn Zagato de Oliveira[3]; Nathalya Cristina de Moraes Roso Mesquita[3]; Natasha Marques Cassani[4]; Igor Andrade Santos[4]; Joyce Villa Verde Bastos Borba[1]; Vinícius Alexandre Fiaia Costa[1]; Bruno Junior Neves[1]; Rafael Victorio Carvalho Guido[3]; Glaucius Oliva[3]; Ana Carolina Gomes Jardim[4]; Luis Octávio Regasini[2]*; Carolina Horta Andrade[1]*

‡ P. R. P. S. Ramos and M. Mottin contributed equally to this work.

***Corresponding authors**
E-mail addresses: carolina@ufg.br (C. H. Andrade), luis.regasini@unesp.br (L.O. Regasini).

[1]LabMol- Laboratory for Molecular Modeling and Drug Design, LabMol, Faculdade de Farmácia, Universidade Federal de Goiás, Goiânia, GO, Brazil

[2]Laboratory of Antibiotics and Chemotherapeutics (LAC), Institute of Biosciences, Humanities and Exact Sciences, São Paulo State University (Unesp), São José do Rio Preto, SP, Brazil

[3]LaBEFar - Laboratory of Structural Biology and Drugs, Institute of Physics of São Carlos, University of São Paulo, São Carlos, Brazil

[4]Laboratory of Antiviral Research, Institute of Biomedical Science, ICBIM, Federal University of Uberlandia, Uberlândia, MG, Brazil

Supplementary Figures

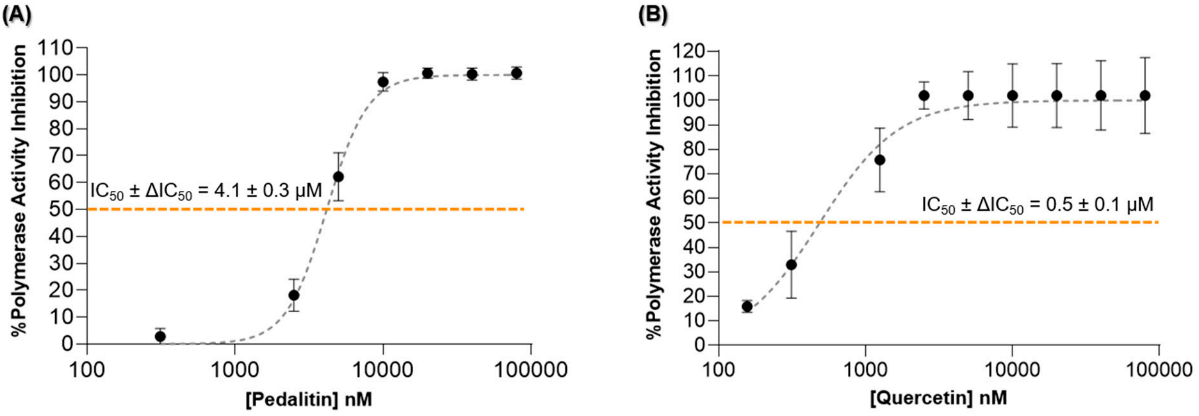

Figure S1. ZIKV NS5 RdRp enzymatic assays. Concentration-response curves adjusted with Hill to determine $IC_{50} \pm \Delta\ IC_{50}$ values for **A)** pedalitin and **B)** quercetin.

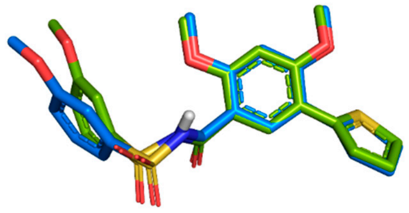

Figure S2. Superposition of the co-crystallized 5-(3-fluorothiophen-2-yl)-2-hydroxy-4-methoxy-N-[4-(trifluoromethyl)benzenesulfonyl]benzamide compound in crystal (C atoms are represented in blue) and the redocking pose (C atoms are represented in green) at the ZIKV NS5 RdRP structure (PDB ID 6LD4 [21]).

Supplementary Tables

Table S1: Docking results for all compounds selected by chemical space analysis

Compound	SMILES	Docking score (Kcal·mol-1)	LE*(Kcal·mol-1·non-hydrogen atom-1)
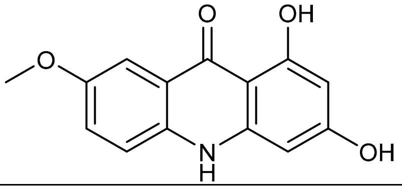	OC1CC2[NH]C3C(C(=O)C2C(O)C1)CC(OC)CC3	-7.92	0.42
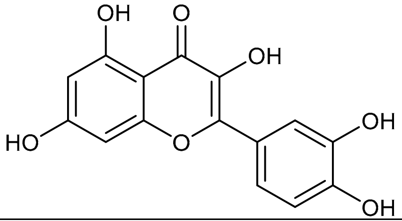	OC1CC2OC(C(C(=O)C2C(O)C1)O)C1C=C(O)C(O)=CC=1	-7.74	0.35
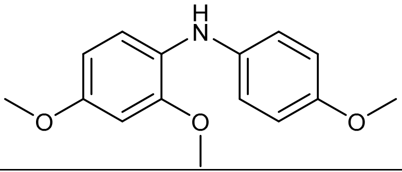	N(C1C(OC)=CC(OC)=CC=1)C1C=CC(OC)=CC=1	-8.12	0.43
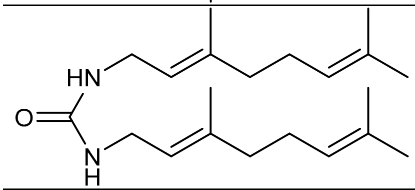	N(CC=C(CCC=C(C)C)C)C(NCC=C(CCC=C(C)C)C)=O	-7.65	0.32
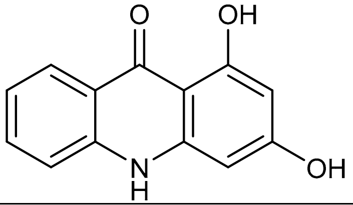	OC1CC2[NH]C3C(C(=O)C2C(O)C1)CCCC3	-7.20	0.42
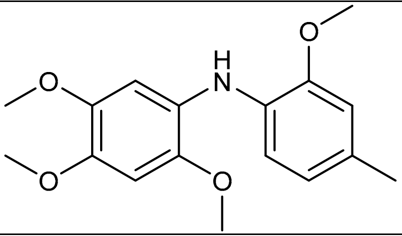	N(C1C(OC)=CC(OC)=C(OC)C=1)C1C(OC)=CC(C)=CC=1	-8.43	0.38

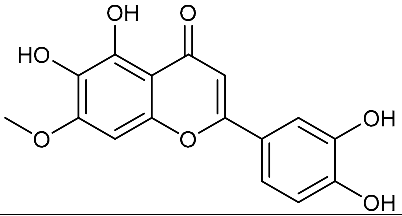	OC1C(O)=CC(C2CC(=O)C3C(CC(C(C3O)O)OC)O2)=CC=1	-7.94	0.35
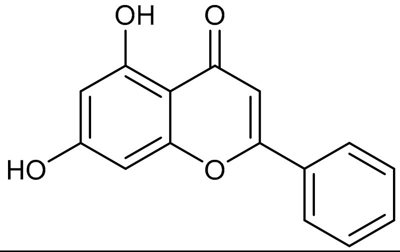	OC1CC2OC(C3C=CC=CC=3)CC(=O)C2C(O)C1	-8.14	0.43
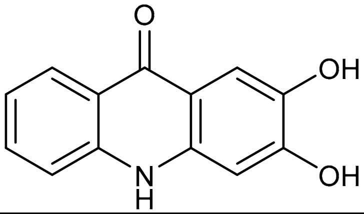	OC1=CC2=C(C=C1O)C(=O)C1=CC=CC=C1N2	-7.01	0.41
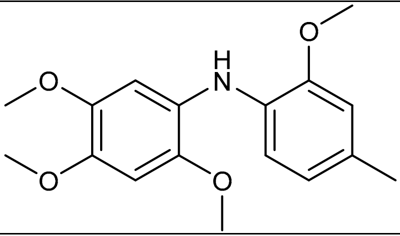	N(C1C(OC)=CC(OC)=C(OC)C=1)C1C(OC)=CC(C)=CC=1	-8.68	0.38
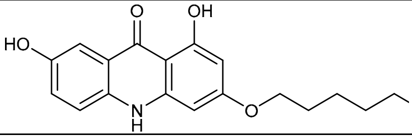	CCCCCCOC1=CC2=C(C(O)=C1)C(=O)C1=CC(O)=CC=C1N2	-8.30	0.35
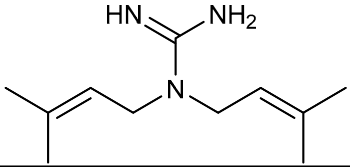	CC(C)=CCN(CC=C(C)C)C(N)=N	-6.62	0.47
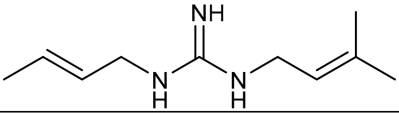	CC(C)=CCNC(=N)NCC=C(C)C	-6.95	0.50
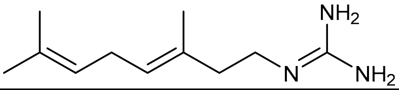	CC(C)=CC\C=C(/C)CCN=C(N)N	-7.38	0.53
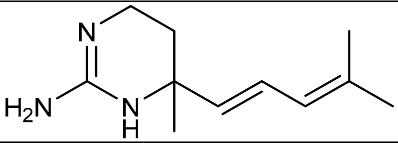	CC(C)=C\C=C\C1(C)CCN=C(N)N1	-7.71	0.55
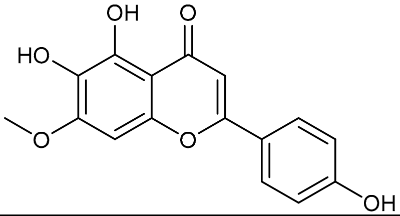	COC1=C(O)C(O)=C2C(=O)C=C(OC2=C1)C1=CC=C(O)C=C1	-8.36	0.38

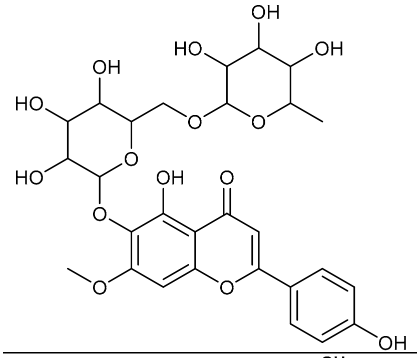	COC1=C(OC2OC(COC3OC(C)C(O)C(O)C3O)C(O)C(O)C2O)C(O)=C2C(=O)C=C(OC2=C1)C1=CC=C(O)C=C1	-7.87	0.18
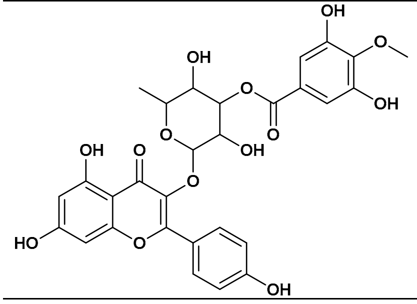	COC1=C(O)C=C(C=C1O)C(=O)OC1C(O)C(C)OC(OC2=C(OC3=CC(O)=CC(O)=C3C2=O)C2=CC=C(O)C=C2)C1O	-7.72	0.18
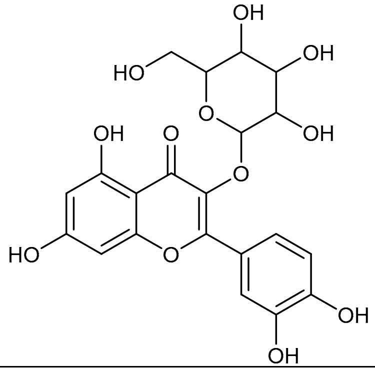	OCC1OC(OC2=C(OC3=CC(O)=CC(O)=C3C2=O)C2=CC(O)=C(O)C=C2)C(O)C(O)C1O	-7.62	0.23
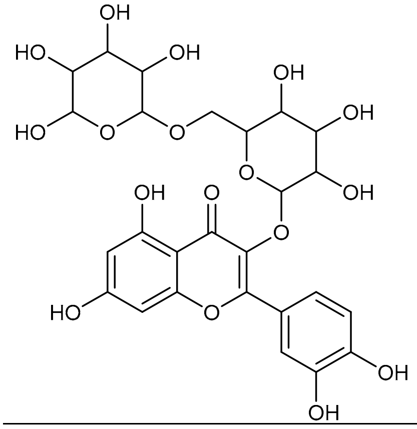	OC1OC(OCC2OC(OC3=C(OC4=CC(O)=CC(O)=C4C3=O)C3=CC(O)=C(O)C=C3)C(O)C(O)C2O)C(O)C(O)C1O	-7.97	0.19
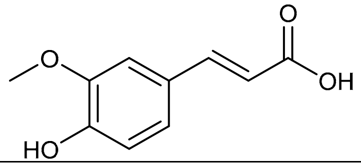	COC1=C(O)C=CC(\C=C\C(O)=O)=C1	-7.54	0.54
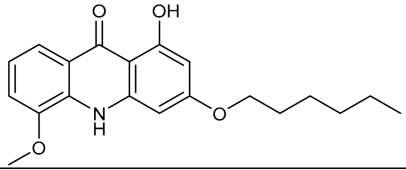	CCCCCCOC1=CC2=C(C(O)=C1)C(=O)C1=CC=CC(OC)=C1N2	-8.72	0.35

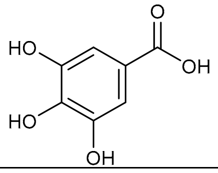	OC(=O)C1=CC(O)=C(O)C(O)=C1	-7.72	0.64
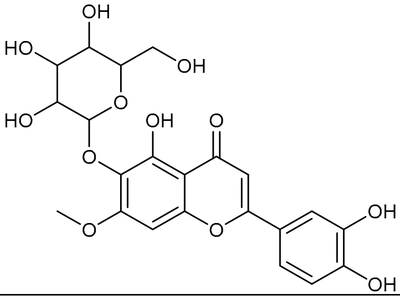	COC1=C(OC2OC(CO)C(O)C(O)C2O)C(O)=C2C(=O)C=C(OC2=C1)C1=CC(O)=C(O)C=C1	-6.99	0.21
